# Set of the data for modeling large-scale coal-fired combined heat and power plant in Kazakhstan

**DOI:** 10.1016/j.dib.2022.108547

**Published:** 2022-08-20

**Authors:** Nurkhat Zhakiyev, Zhuldyz Sotsial, Aldiyar Salkenov, Ruslan Omirgaliyev

**Affiliations:** Astana IT University, 55/11 Mangilik Yel ave., 010000 Nur-Sultan, Kazakhstan

**Keywords:** Coal CHP, Combined heat and power, Electricity and heat production, Techno-economic, unit commitment, Kazakhstan, RCU, reduction-cooling units, CHPP, combined heat and power plant, TO, the ambient air temperature, DemEH, Electricity and heat demand, DH, heat demand, DS, steam demand, DE, Electricity demand, EB, installed boilers, ET, installed turbines

## Abstract

This data article provides information on input data to represent the operational processes of two combined heat and power plants (CHPP) in Kazakhstan. The presented data in this article are related to the research article “Efficient planning of energy production and maintenance of large-scale combined heat and power plants” (G. Kopanos et al. 2018). This set of data is helpful in modelling two different cases of the Industrial coal-fired power plants installed in 1970-1990 during the Soviet Union period. This data article presents technical characteristics of boiler equipment, turbine units, steam parameters, the demand curve for heat energy and electricity, and their correlation with ambient temperature. Also provided detailed technical and economic parameters of considered conventional Coal-Fired CHP plants. The dataset cases for two CHPP is made publicly available to allow researchers to test novel mathematical models and modeling methods on real industrial data. The dataset cases are useful for research tasks in advanced unit commitment problems coupled with maintenance scheduling.

**Specifications Table**

**Table:** 

Subject	Energy Engineering and Power Technology
Specific subject area	Technical and Economic Data on the Coal-Fired Combined Heat and Power Plant
Type of data	Tables, graphs, figures
How data was acquired	Extensive desktop research for compilation the set of data from various sources in literature, including translation from Russian, expert knowledge, technical reports, scheme of CHPP, initial documentations of the boilers and turbines
Data format	Raw and processed
Description of data collection	Data on power plant efficiency and age structure was retrieved from public sources and reconfirmed with expert knowledge. Operational data from metering system indexed by units, depersonalized, and generalized with additional calculations of specific parameters. Prepared as an input file ready for applied research.
Data source location	Karaganda, Kazakhstan (N 49.80468; E 73.10938), Pavlodar, Kazakhstan (N 52.2873; E 76.9674),
Data accessibility	Data is with this article and included in the accompanying excel file and https://data.mendeley.com/datasets/3tprt2ct2n/2
Related research article	G. Kopanos, O.C. Murele, J. Silvente, N. Zhakiyev, Y. Akhmetbekov, D. Tutkushev, Efficient planning of energy production and maintenance of large-scale combined heat and power plants, Energy Conversion and Management, 169 (2018) 390-403. https://doi.org/10.1016/j.enconman.2018.05.022

## Value of the Data

• The data serves for a better understanding of the technical and economical characteristics of the most generation units installed in the Kazakhstan energy system;
• The experimental data reported in this article will enhance further research in the field of operational research in energy economics, especially in the area of modeling unit commitment and maintenance scheduling;
• Researchers and engineers in power modeling can benefit from these data by modeling power plant generation;
• The data can be used for models in operational research with different energy vectors;
• An extensive data analysis of historical data has been performed to prepare the necessary input data;
• The presented data is valuable input data for energy and heat systems with different consumers and types of energy (heat, electricity, and steam).

## 1 Data Description

The data file contains seven spreadsheets for 2 different cities (two cases) and it is structured as follows: The spreadsheets “DemEH” provide information on demand profiles (DH, DS and DE in the spreadsheet) in City A and City B (Case 1 and Case 2) as well temperature profiles of these cities. The spreadsheets “MaxMin” contain maximum and minimum installed capacities of the boilers and turbines. The spreadsheets “UnifEff” contain monthly efficiency rates of the boilers (EB in the spreadsheet) and turbines (ET in the spreadsheet). The spreadsheet “Turbine” useful for both cases, contains data on lower and upper bounds of steam consumption and heat workload with respect to various temperature features for heating supply. In both Cases (City 1 and City 2), spreadsheet DemEH1 and DemEH2 contains “OwnUse” parameter in %, which describes how many portions of daily or hourly generated energy was spent to their own needs. The CHP plants have heat, steam and electricity loads, which were provided in these spreadsheets.

The spreadsheet “DemEH Case 2” consists of the daily heat demand in GCal for the investigated year. The table includes the peak seasons of CHPP productivity and minimal loading periods in the year. The power plant's performance is highly influenced by the ambient air temperature (TO in the spreadsheet) due to the increased consumption of coal for heating residential buildings. Statistically, the range of the temperature in Kazakhstan is from –40 to +40 degrees. In the given period the lowest temperature was minus 37 and the highest is plus 25.

The spreadsheet “Turbine” contains data on lower and upper bounds of heat and electricity energy production that depends on the energy of the steam with respect to various temperature features for heating supply through heat networks.

All these dataset cases are useful for research tasks in advanced unit commitment problems coupled with maintenance scheduling and to estimate the seasonal gross efficiency of CHP plants.

## 2 Experimental Design, Material and Methods

### 2.1 Dataset acquisition

Large capacities of combined heat and power (CHP) plants are a distinctive feature of the power system of Kazakhstan. The wide deployment of CHP plants was dictated by Soviet central planning, and it was convenient utilization of low-grade heat for district heating due to the cold climate and abundance of coal reserves. The aim of the paper is to explain the input data (excel file), which was used for a detailed analysis of combined heat and power plants and their demand profiles in different cities of Kazakhstan [1]. The data file (Datasets for Case 1 and Case 2) associated with this article can be found in the online version at [2]. A combination of quantitative and qualitative approaches was used in the data analysis of demand variations and distributions, technical and consumption parameters to provide optimal solutions and increase the efficiency of CHPPs using the proposed optimization model consisting: (i) unit commitment constraints for boilers and turbines; (ii) bounds on the operating levels for boilers and turbines within desired operating regions; (iii) extreme operating regions for turbines; (iv) energy balances for turbines; (v) total electricity and heat balances for electricity and heat network; and (vi) maintenance tasks for units within given time-windows [3], [4].

**Fig. 1:**
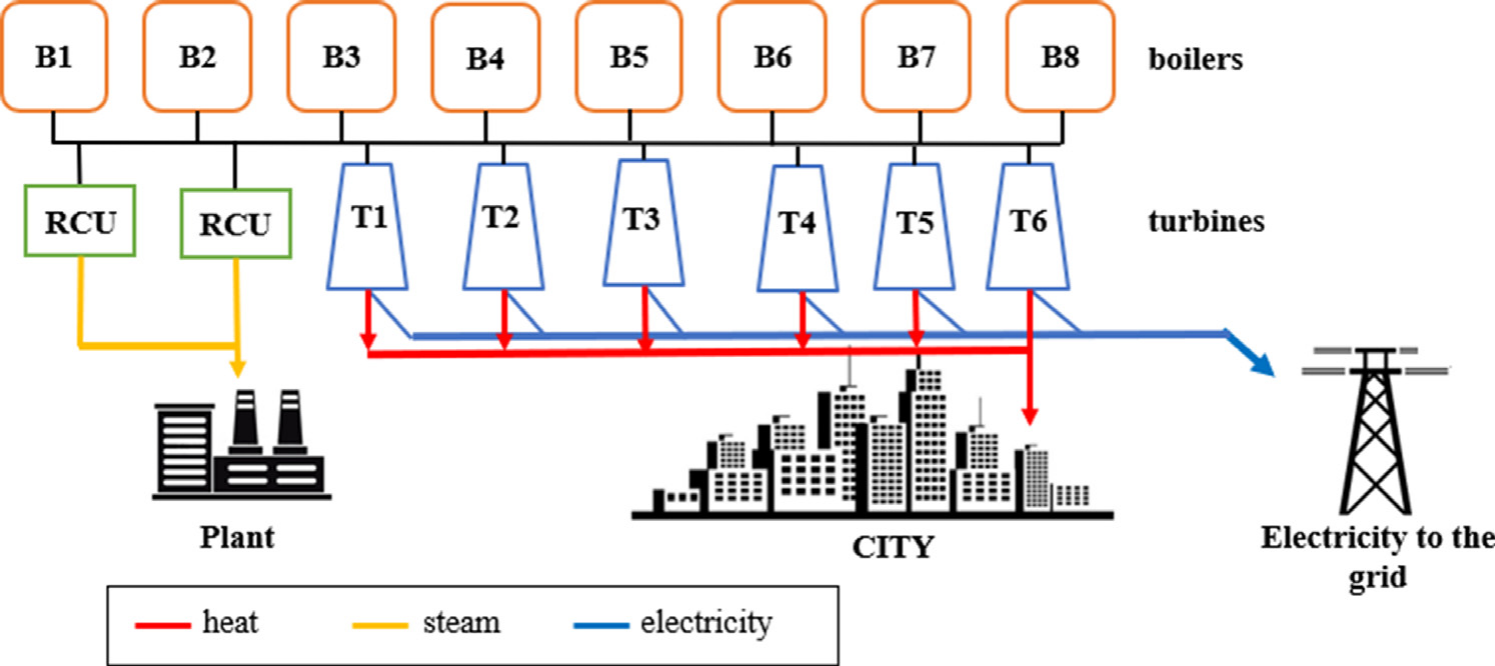
Main scheme-structure of the CHPP for Case 2 (with industrial steam consumption). There, brown boxes – boilers, blue shapes – turbines, green-RCU – reduction-cooling units (Developed by authors).

The acquired data provides details on CHPPs units as well as demand time series, installed unit efficiencies, its capacities, and age structure was retrieved from public sources and reconfirmed with expert knowledge. It combines a large set of data points from various sources and presents it in an easily accessible way to a global English-speaking audience. In the quickly growing research field of operational research in energy economics, this data-in-brief sets the necessary starting point to explore old CHPPs based on fossil fuel installed in the post-Soviet Union area and continues to operate. This set of data is helpful in modelling two different cases of the Industrial coal-fired power plants operating in Kazakhstan. They are installed in the Soviet Union period for 1970-1990 years. This data article presents technical characteristics of boiler equipment, turbine units, steam parameters, the demand curve for heat energy and electricity, and their correlation with ambient temperature [5]. The dataset could be used for the comparative assessment of the environmental, economic and energy parameters of the different types of CHP [6,7] (Fig. 1).

The overall structure of the data article takes the form of three main topics, including:

• Technical parameters for CHPPs for Case A and Case B;
• Time series of the energy demand in the cities and ambient temperatures for City A and City B;
• Maximum and minimum installed capacities of boilers and electricity turbines;
• Unit efficiency of the installed boilers (EB) and turbines (ET).

The dataset contains annual information between 1 January to 31 December: Steam Pressure (kg/cm²), Steam Temperature (°C) and Steam Flow Rate (t/h). They were initially analysed from averaged hourly resolution. For comparative investigation during the whole year, it was necessary to add the environmental temperature (°C) of a city to the time series.

### 2.2 Dataset processing

For the next thermodynamical analysis, it is necessary to find Enthalpy. Using Steam Temperature (°C) and Steam Pressure (kg/cm²), we found the Enthalpy. To calculate the Enthalpy, we used the following formula:

Superheated vapor up to 1073.15 K temperature.

Specific enthalpy h (Eq.1):

(1) $$h=g-T{\left(\partial g\;/\;\partial T\right)}_{p}$$

where, T is Absolute temperature, ɡ is Specific Gibbs free energy.

To find for the study of Energy Consumption (kCal) used (Eq. 2) included Enthalpy (kCal/kg) and Steam Flow Rate (t/h).

(2) E=h·Q

where, E is Power (MW), h is Specific Enthalpy (kCal/kg), Q is Flow Rate (t/h).

### 2.3 Methods for preparing input data for modelling

**Fig. 2:**
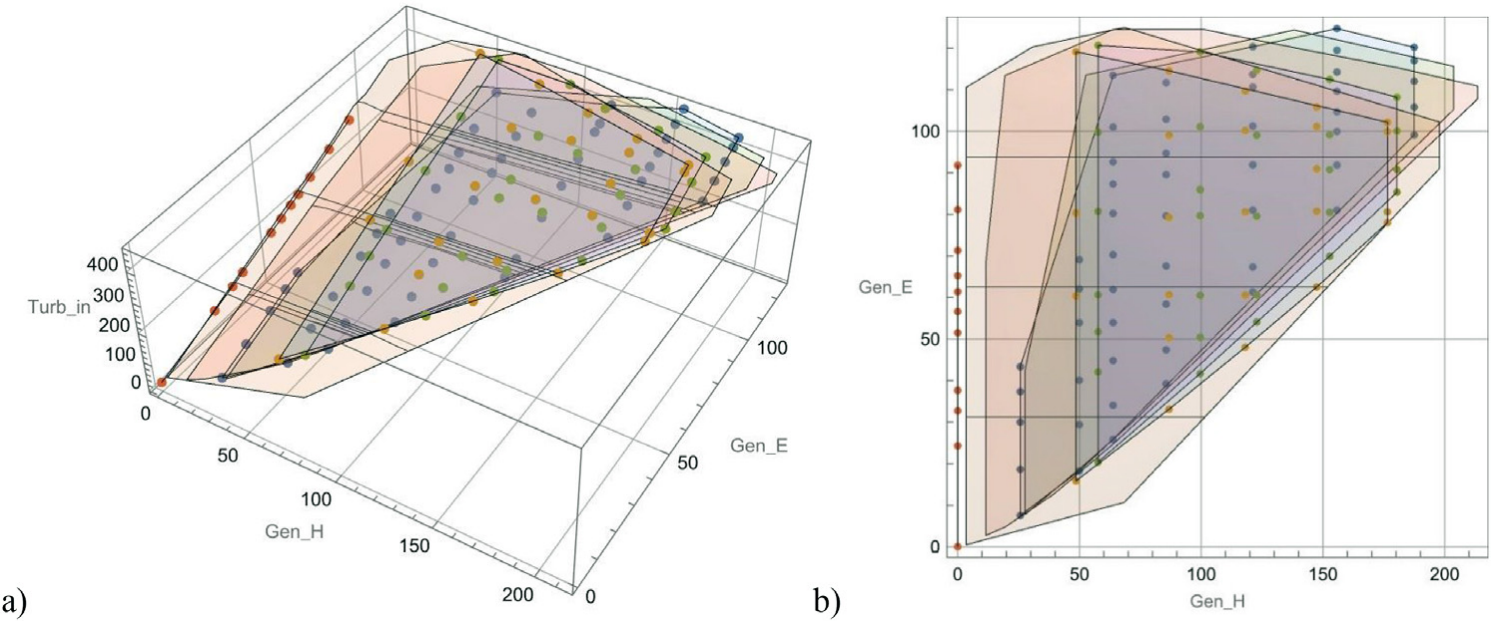
Dependence between the steam supplied to the turbine head and the electrical and heating outputs at different temperature conditions: a) a three dimensional view – inlet steam energy in MW, electric power (MW) and heating capacity (MW); b) a two dimensional top view – dependence between electric power (MW) and heating capacity (MW). (Developed by authors)

Optimization of the operation of a CHHP is an essential technical and economic task aimed at improving the efficiency of using natural resources that serve as fuel for CHPP and increasing the economic efficiency of the plant's operation in the electricity and heat market. For modelling, the dependence between the steam energy supplied to the turbine head and the electrical and thermal loads at different temperature conditions for the heating network from 75°C to 125°C were studied and extracted the data of dependences (Fig. 2).

In CHP processes based on steam turbines, there is a trade-off between power output lost and useful heat recovery making the full load line downward sloping [8]. Fig. 2 indicates the visualized spreadsheet “Turbine”, with three dimensional lower and upper constraints of heat and electricity energy production that depends on the energy of the steam with respect to various temperature features for heating supply regimes through heat networks. Fig. 2a is a conversion dependence of steam energy recalculated into MW between the electric power in MW and a heating capacity in MW. Fig. 2b two-dimensional dependence between electric power and heating capacity in MWs. Moreover, in two-dimensional dependence, we have linear relationship between electric power (x) and heating capcacity (y). That linear dependence can be described as y = kx+b or in Nomenclature Description, which was provided previously, you can find the following formulas:

H=k_HQ*Q+b_HQ

(3) E=k_EH*Q+b_EH

Where H – heating capacity, E – electric energy, k_HQ and b_HQ – coefficients of linear equations, which were obtained experimentally in programming tool.

## 3 Data on Electricity, Heat, and Steam Demands

The main parameters of boilers are shown in Table 1, which is provided in the spreadsheet “MaxMin” for Case 1 as the minimum and maximum steam production capacity for boilers in MW and their seasonal efficiency in %. All these data are used to estimate the seasonal gross efficiency of boilers.

**Table 1:** Minimum and maximum steam production capacity for boilers in MW and their seasonally efficiency in % (For Case 1).

Boiler	B1	B2	B3	B4	B5	B6	B7	B8
Minimum capacity, $Q_{i}^{Bmin}$ (MW)	145.3	145.3	145.3	145.3	145.3	145.3	145.3	231.3
Maximum capacity, $Q_{i}^{Bmax}\;$(MW)	290.6	290.6	290.6	290.6	290.6	290.6	290.6	464.9
Efficiency in summer, $\eta_{i,t}\;$(%)	86.30	86.40	86.50	87.69	87.60	87.80	87.90	90.15
Efficiency in winter, $\eta_{i,t}\;$(%)	87.20	87.50	87.90	88.08	88.70	88.80	88.90	91.41

**Fig. 3:**
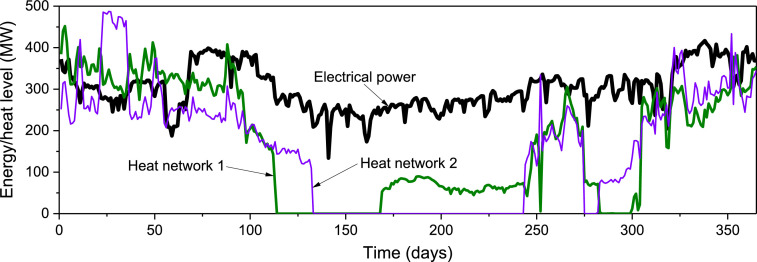
Annual electrical power and heat demand profiles for Case 1. Reprinted from [11]. Copyright permission received from Elsevier.

**Fig. 4:**
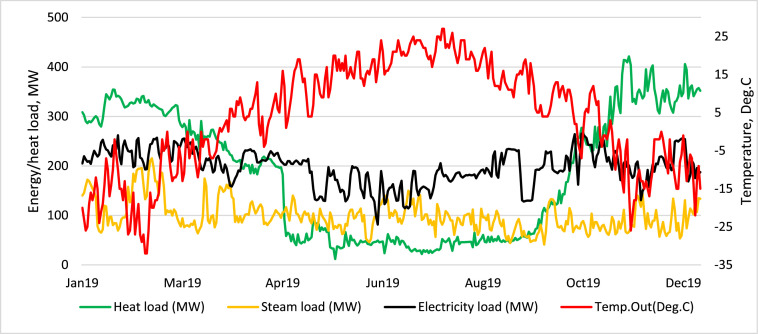
Annual electrical power, steam and heat demand profiles for Case 2.

Demand profiles for electrical power and heat networks are displayed in Figs. 3 and 4. Data of ambient temperature were obtained from the national hydrometeorological service of Kazakhstan and Wolfram Mathematica [9,10] in hourly resolution, after recalculation as an average daily temperature and further transformation them into heat supply commitment modes. Fig. 4 shows that the uppermost heat demand accounts for January. The Demand for CHPPs emerges from residential consumption and the aluminium plant where the heat goes to urban needs and the steam to the plant.

Tables 2 and 3 display the minimum and maximum electricity and heat capacities for the turbines in MW (Case 1). The maximum installed heat capacity in ideal conditions for turbines T1-T4 is 203.4 MW, for T5 is 218.5 MW, and for T6 is 197.6 MW. The average efficiency for each month is considered and Table 3 displays the maximum heat capacity for each turbine at each time period.

**Table 2:** Installed minimum and maximum capacity for turbines (modified from [1]. Copyright permission received from Elsevier).

Turbine	Minimum electric capacity, $E_{i}^{Tmin}$ (MW)	Maximum electric capacity, $E_{i}^{Tmax}$ (MW)	Maximum heat capacity, $Q_{i,t}^{Tmax}$ (MW)
T1-T4	66.0	110.0	203.4
T5	72.0	120.0	218.5
T6	66.0	110.0	197.6

**Table 3:** Seasonally electrical efficiency and maximum heat capacity of turbines.

Month	Electrical efficiency, $\eta_{i,t}$,of T1-T6 (%)	Maximum heat capacity, $Q_{i,t}^{Tmax}$, of T1-T4, T6 (MWh/day)	Maximum heat capacity, $Q_{i,t}^{Tmax}$, of T5 (MWh/day)
m1	60	5577.6	6972.0
m2	60	4182.0	6525.8
m3	55	3004.6	2581.2
m4	50	3004.6	503.8
m5	45	182.5	0.0
m6	35	182.5	0.0
m7	35	182.5	0.0
m8	35	182.5	0.0
m9	40	182.5	0.0
m10	50	2314.8	1941.7
m11	55	2683.2	3917.9
m12	60	4182.0	4182.0

This data article presents technical characteristics of boiler equipment, turbine units, steam parameters, the demand curve for heat energy, electricity, and ambient temperature. The dataset cases for two CHPP is made publicly available to allow researchers to test their mathematical models and modeling methods on real industrial data for unit commitment problems coupled with maintenance scheduling.

## 4 Nomenclature of the Parameters

**Table:** 

iϵI	Processing units
$i\epsilon I^{T}$	Turbine units
$i\epsilon I^{B}$	Boiler units
$E_{i}^{Tmax}$	Maximum amount of energy to be generated in turbine $i\epsilon I^{T}$
$E_{i}^{Tmin}$	Minimum amount of energy to be generated in turbine $i\epsilon I^{T}$
$Q_{i}^{Bmax}$	Maximum amount of heat to be generated in boiler $i\epsilon I^{B}$
$Q_{i}^{Bmin}$	Minimum amount of heat to be generated in boiler $i\epsilon I^{B}$
$Q_{i}^{Tmax}$	Maximum amount of heat to be generated in turbine $i\epsilon I^{T}$
$\eta_{i,t}$	Efficiency of turbine $i\epsilon I^{T}$

## Acknowledgment

This research has been funded by the Science Committee of the Ministry of Education and Science of the Republic of Kazakhstan (“Modeling and machine learning methods for optimal planning of the generating equipment of CHP" [Grant No. AP09563335]); (N.Z. and R.O.) acknowledges the Frontiers Champions programme of Royal Academy of Engineering (FC-2122-2-39) for developing an academic network. We would like to thank Dr. Daniel Friedrich for providing insightful feedback on an early draft of this data article.

## CRediT authorship contribution statement

**Nurkhat Zhakiyev:** Writing – original draft, Resources, Supervision. **Zhuldyz Sotsial:** Validation, Visualization, Writing – review & editing, Project administration. **Aldiyar Salkenov:** Software, Data curation, Writing – review & editing. **Ruslan Omirgaliyev:** Methodology, Validation, Formal analysis, Writing – review & editing.

## Declaration of Competing Interest

The authors declare that they have no known competing financial interests or personal relationships that could have appeared to influence the work reported in this paper. The funders had no role in the design of the study; in the collection, analyses, or interpretation of data.

## Data Availability

Data for Modeling Large-scale Coal-Fired CHP Plant in Kazakhstan: Two Cases (Original data) (Mendeley Data).
